# Supplementary material for: Vector-Borne Pathogens in Stray Cats in Eastern Germany (Thuringia)
Source: Animals (Basel). 2023 Aug 10;13(16):2574. doi: 10.3390/ani13162574 (PMC10451234; doi:10.3390/ani13162574)
Supplement: Supplementary file 1 [file animals-13-02574-s001.zip › animals-2510704-supplementary.pdf]

animals

MDPI

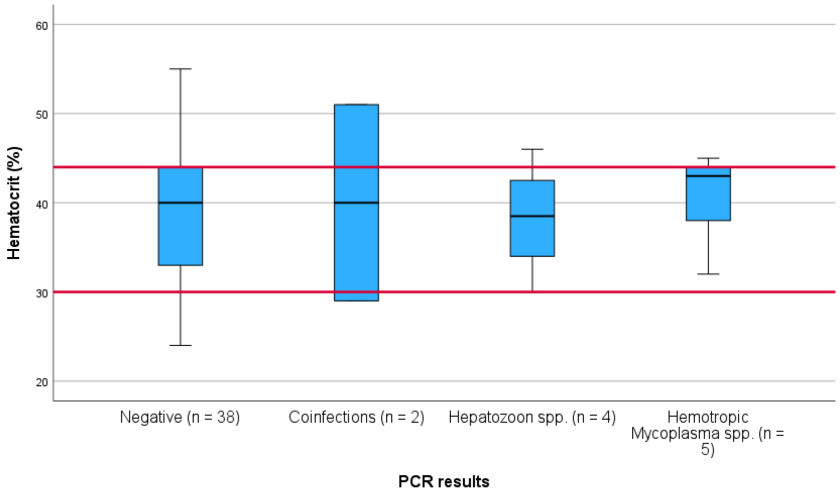

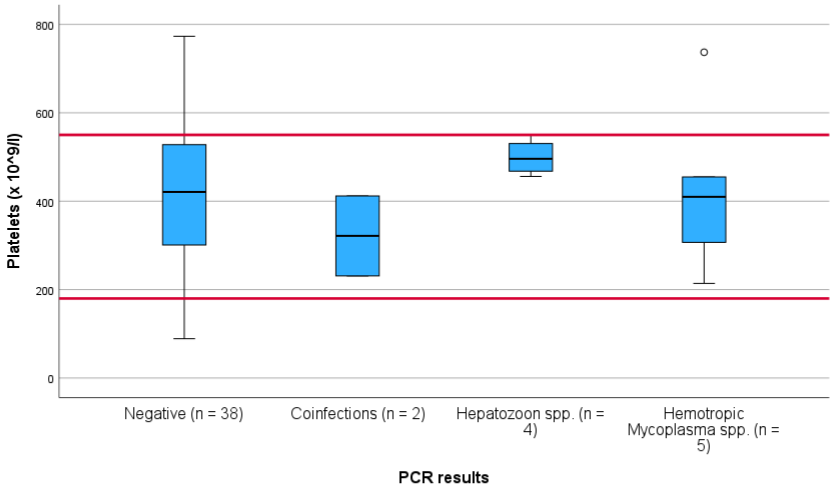

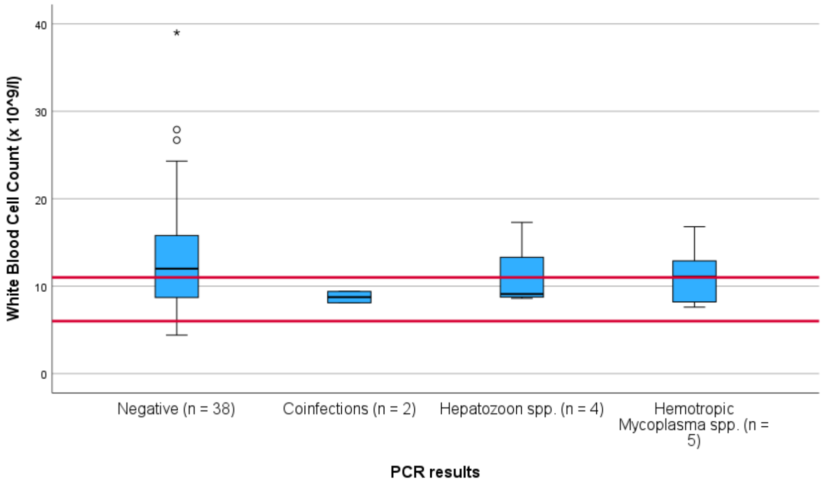

Suppl. figure S1. Results of selected hematological analysis in 49 stray cats in Germany sorted by negative PCR results and positive results for coinfections (*Anaplasma phagocytophilum* and *Hepatozoon* spp., *Anaplasma phagocytophilum* and hemotropic *Mycoplasma* spp.), *Hepatozoon* spp., and hemotropic *Mycoplasma* spp.

Red lines: reference interval of the LABOKLIN laboratory; ° = mild outliers (values that are more than 1.5 x interquartile range below Q1 or above Q3 in the boxplot); * = extreme outliers (values that are more than 3.0 x interquartile range below Q1 or above Q3 in the boxplot)

Suppl table S1. Hematological and biochemical parameters in 50 apparently healthy stray cats in Thuringia (Germany) presenting numbers of cats with elevated and decreased parameters according to the reference interval (RI) by the laboratory in cats tested negative, in cats with coinfections, and in cats tested positive for *Hepatozoon* spp. and hemotropic *Mycoplasma* spp. by Polymerase Chain Reaction (PCR)

Parameter	RI	Negative (hematology n = 38, biochemistry n = 39)	Coinfections (n = 2)	*Hepatozoon* spp. (n = 4)	Hemotropic *Mycoplasma* spp. (n = 5)	P	Total (hematology n = 49, biochemistry n =50)
**Hematology**[A]							
Erythrocytes (x 10^{12}/l)	5.0 – 10.0	5.3 – 11.1 (8.8)	7.1 – 11.0 (9.0)	7.3 – 9.7 (9.2)	7.2 – 11.0 (8.3)	0.997	5.27 - 11.1 (8.76)
Hemoglobin (g/dl)	90 – 150	73 – 162 (123.5)	110 – 165 (137.5)	99 – 140 (130)	89 – 152 (129)	0.889	73 – 165 (126)
Hematocrit (%)	30 – 44	24 – 55 (40)	29 – 51 (40)	30 – 46 (39)	32 – 45 (43)	0.959	24 – 55 (40)
Reticulocytes (/µl)	< 60.0	3.0 – 127.5 (20.8)	8.5 – 38.6 (23.6)	11.6 – 32.0 (19.2)	12.2 – 53.7 (23.1)	0.920	3.0 – 127.5 (21.6)
Reticulocyte hemoglobin content (pg)	< 11.5	13.6 – 19.8 (16.8)	16.9 – 19.7 (18.3)	15.4 – 16.3 (15.9)	14.6 – 19.2 (16.2)	0.188	13.6 – 19.8 (16.5)
Leukocytes (x 10^9/l)	6.0 – 11.0	4.4 – 39.0 (12.0)	8.1 – 9.4 (8.8)	8.6 – 17.3 (9.1)	7.6 – 16.8 (11.1)	0.536	4.4 – 39.0 (10.9)
Segmented neutrophils (x 10^9/l)	3.0 – 11.0	2.6 – 33.2 (8.2)	5.3 – 8.4 (6.9)	5.2 – 14.4 (5.9)	5.2 – 10.8 (8.5)	0.623	2.6 – 33.2 (10.9)
Eosinophils (x 10^9/l)	0.04 - 0.6	0.1 – 2.0 (0.7)	0.4 – 0.5 (0.5)	0.4 – 0.9 (0.6)	0.0 – 1.2 (0.3)	0.232	0.0 – 2.0 (0.6)
Lymphocytes (x 10^9/l)	1.0 – 4.0	1.0 – 6.4 (2.0)	0.6 – 2.1 (1.4)	2.0 – 2.7 (2.3)	1.5 – 5.0 (2.3)	0.604	0.6 – 6.4 (2.1)
Monocytes (x 10^9/l)	0.04 -0.5	0.1 – 1.1 (0.3)	0.1 – 0.2 (0.2)	0.2 – 0.4 (0.3)	0.1 – 0.3 (0.2)	0.280	0.1 – 1.1 (0.3)
Basophils (x 10^9/l)	< 0.04	0.0 – 0.1 (0.0)	0.0	0.0	0.0	0.898	0.0 – 0.1 (0.0)
Platelets (x 10^9/l)	180- 550	89 – 773 (421)	231 – 412 (322)	456 – 549 (496)	214 – 737 (410)	0.497	89 – 773 (447)
**Biochemistry**[B]							
Sodium (mmol/l)	145 – 158	145 – 156 (150)	149 – 152 (151)	147 – 152 (150)	150 – 152 (150)	0.810	145 – 156 (150)
Potassium (mmol/l)	3.0 – 4.8	3.8 – 5.8 (4.6)	3.9 – 4.6 (4.3)	4.4 – 5.3 (5.0)	4.3 – 5.9 (4.6)	0.475	3.8 – 5.9 (4.6)
Glucose (mmol/l)	3.1 – 6.9	2.7 – 15.7 (7.0)	8,8 – 11.0 (9.9)	6.5 – 10.9 (8.9)	5.8 – 10.6 (7.2)	0.769	2.7 – 15.7 (7.2)
Fructosamine (mmol/l)	< 340	208 – 341 (248)	234 - 277 (255)	198 – 231 (222)	207 – 271 (240)	0.074	198 – 341 (246)
Creatinine (µmol/l)	< 168	36 – 119 (87)	83 – 91 (87)	69 – 104 (87)	82 – 108 (90)	0.763	36 – 119 (87)
Urea (mmol/l)	5.0 – 11.3	4.3 – 12.2 (7.9)	8.2 – 8.6 (8.4)	7.0 – 7.4 (7.2)	6.6 – 11.3 (8.3)	0.491	4.3 – 12.2 (7.7)
Alanine transaminase (U/l)	< 70	23 – 114 (56)	31 – 39 (35)	24 – 39 (31)	32 – 69 (51)	**0.019**	23 – 114 (46)
Aspartate transaminase (U/l)	< 30	9 – 50 (19)	12 – 15 (14)	9 – 14 (12)	16 – 31 (21)	0.060	9 – 50 (18)
Alkaline phosphatase (U/l)	< 140	10 – 124 (33)	12 – 17 (15)	17 – 54 (21)	17 – 40 (31)	0.124	10 – 124 (31)
Glutamate dehydrogenase (U/l)	< 6.0	0.3 – 8.2 (1.3)	0.8 – 1.4 (1.1)	0.4 – 3.1 (1.2)	0.6 – 1.7 (1.1)	0.793	0.3 – 8.2 (1.3)
Bilirubin (µmol/l)	< 3.4	0.1 – 2.9 (0.7)	0.7 – 0.7 (0.7)	0.4 – 1.1 (0.7)	0.4 – 0.7 (0.5)	0.513	0.1 – 2.9 (0.7)
Creatine kinase (mmol/l)	< 130	54 – 1442 (234)	171 – 191 (181)	84 – 201 (112)	118 – 585 (207)	0.329	54 – 1,442 (201)
Calcium (mmol/l)	2.3 – 3.0	2.2 – 2.8 (2.4)	2.3 – 2.5 (2.4)	2.2 – 2.5 (2.4)	2.2 – 2.6 (2.4)	0.652	2.2 – 2.8 (2.4)
Phosphorus (mmol/l)	0.8 – 1.9	1.0 – 3.1 (2.0)	1.7 – 1.9 (1.8)	1.5 – 2.5 (2.1)	1.4 – 2.4 (1.8)	0.726	1.0 – 3.1 (1.9)
Total protein (g/l)	57.0 – 94.0	62.4 – 91.7 (72.6)	76.9 – 77.8 (77.4)	71.7 – 84.9 (81.4)	66.9 – 80.7 (73.7)	0.201	62.4 – 91.7 (73.4)

Albumin (g/l)	26.0 – 56.0	29.6 – 45.2 (36.3)	29.2 – 42.0 (35.6)	34.7 – 38.1 (36.4)	32.3 – 39.9 (34.6)	0.989	29.2 – 45.2 (36.1)
Globulin (g/l)	< 55.0	26.0 – 56.9 (38.0)	34.9 – 48.6 (41.8)	37.0 – 48.4 (44.2)	27.3 – 41.4 (40.8)	0.399	26.0 – 56.9 (38.2)
DGGR lipase (U/l)	< 26.0	7.5 – 30.0 (15.8)	7.2 – 12.0 (9.6)	9.3 – 15.7 (11.0)	10.9 – 16.0 (15.4)	0.277	7.2 – 30.0 (14.0)
Iron (µmol/l)	8.0 – 31.0	5.3 – 32.5 (13.7)	13.6 – 21.6 (17.6)	13.4 – 15.0 (14.5)	7.7 – 15.9 (11.1)	0.523	5.3 - 32.5 (13.6)
T4 (µg/dl)	0.9 – 2.9	0.8 – 2.6 (1.5)	1.3 – 1.4 (1.4)	1.2 – 1.7 (1.6)	1.0 – 1.4 (1.3)	0.148	0.8 – 2.6 (1.5)

[A]Sysmex XN-V, Sysmex Deutschland, Germany; [B]Cobas 8000, Roche, Germany

P = calculation of statistical significance ($P < 0.05$) for hematological and biochemical parameters between cats tested negative, cats with coinfections, cats tested positive for *Hepatozoon* spp., and cats tested positive for hemotropic *Mycoplasma* spp. using Kruskal-Wallis testing
